# Predicting steroid-induced osteonecrosis of the femoral head: role of lipid metabolism biomarkers and radiomics in young and middle-aged adults

**DOI:** 10.1186/s13018-024-05245-2

**Published:** 2024-11-13

**Authors:** Daqi Jia, Yue Zhang, Huaqiang Li, Chunfang Guo, Yipeng Wu, Xiangwen Shi, Li Yang, Jieyu Mo, Xia Liu, Yongqing Xu

**Affiliations:** 1 Department of Pathology, Affiliated Banan Hospital of Chongqing Medical University, Longzhouwan Street, Yunan District, Chongqing, 401320 China; 2 Kunming Medical University, No. 1168, Chunrong West Road, Yuhua Street, Chenggong District, Kunming, 650500 China; 3 Department of Orthopaedics, 920th Hospital of the Joint Logistics Support Force of the Chinese People’s Liberation Army, 212 Daguan Road, Xishan District, Kunming, 650032 China

**Keywords:** Predictive, Preventive and personalized medicine, Steroid-induced osteonecrosis of the femoral head, Radiomics

## Abstract

**Background:**

Femoral head necrosis is a common orthopedic disease that results in significant physical disability in patients. Early prediction and diagnosis of steroid-induced osteonecrosis of the femoral head (SONFH) are crucial for the prevention and treatment of this condition.

**Methods:**

In this study, initial CT images and clinical data of patients with SONFH, admitted from January 2019 to December 2022, were collected. Patients were grouped as follows: (1) those diagnosed with SONFH at the initial diagnosis (control group), and (2) those with high-risk factors but no symptoms at first diagnosis, who developed SONFH two years later (experimental group). CT imaging histological features, clinical characteristics, and transcriptome screening for differentially expressed genes, pathway enrichment, and immune infiltration analyses were performed.

**Results:**

Significant differences were found in triglyceride (TG) levels between the training and validation groups. Age, sex, alkaline phosphatase (ALP), and hemoglobin levels differed between the training and internal validation groups, while HDL and red blood cell counts varied between the training and external validation groups. Univariate analysis showed that age, TG, HDL, and Radiomics scores influenced SONFH, while multivariate analysis revealed TG, HDL, and Radiomics scores were closely related to SONFH. Transcriptomic analysis showed associations with sphingolipid and adipocyte signaling pathways, along with immune cell involvement, linking SONFH to lipid metabolism and atherosclerosis.

**Conclusions:**

These findings indicate a significant association between steroid-induced osteonecrosis of the femoral head and age, with TG and HDL serving as indicators of lipid metabolism closely correlated with the occurrence of SONFH. Radiomics scores were also found to correlate with SONFH occurrence, supported by transcriptomic and CT imaging findings. However, this study has limitations, including its retrospective design and a relatively limited sample size, which may impact the generalizability of the results. Further prospective studies with larger, more diverse populations are needed to validate and enhance the predictive model.

**Supplementary Information:**

The online version contains supplementary material available at 10.1186/s13018-024-05245-2.

## Background

Steroid-induced osteonecrosis of the femoral head (SONFH) is a progressive degenerative ailment of the hip [1–3]. It manifests through femoral head collapse, acetabular degeneration, and secondary arthritis [4, 5]. Nontraumatic necrosis of the femoral head is often caused by alcohol and steroids, but its cause remains uncertain in some patients. The clinical manifestation of this condition emerges as hip discomfort accompanied by movement restrictions [6, 7]. In recent decades, the annual number of confirmed new cases has ranged from 150,000 to 200,000 in China and from 20,000 to 30,000 in the United States [8]. Frequently, in individuals under 50 years of age, necrosis of the femoral head is more common in males than females [9, 10]. Typically, initiated bilaterally, this disorder manifests as extensive necrosis, swift progression, and marked disability [11], greatly impairing patients’ quality of life. Diagnostic techniques include X-rays, computed tomography (CT), and magnetic resonance imaging (MRI). Successful treatment is determined by precise staging [12]. Moreover, early prevention and diagnosis prove advantageous in preventing disease progression. Thus, early disease prediction emerges as paramount.

Imaging histology stands as an emerging domain within the medical realm. Its essence lies in the augmentation of medical imaging precision through quantitative assessment of data, accomplished via automated or semiautomated software [13]. In the realm of visualizing ailments, CT scanning plays a pivotal role. It aptly reveals lesion extent, spatial orientation, and recovery aspects. Furthermore, it discerns stages II and III, although its capacity for diagnosing stage I SONFH remains limited [14]. The integration of imaging histology with CT images is highly important for predicting necrotic femoral head afflictions in high-risk individuals. In the present study, the control cohort included patients with nontraumatic femoral head osteonecrosis. The experimental assemblage comprised patients marked by high-risk attributes (as delineated in Table 1) and diagnosed with nontraumatic femoral head osteonecrosis two years later. Extracted CT images were subjected to imaging histology analysis, paving the way for an osteonecrosis prediction model tailored to high-risk clusters. Effectively predicting the incidence of femoral head necrosis can reduce the need for femoral head surgery.

**Table 1:** Clinical features of patients in each cohort

Characteristics	Training cohort (*N* = 216)	Internal validation cohort (*N* = 72)	External validation cohort (*N* = 42)
Age (years)	44(38–50)	44.5(36–52)	45.5(40–52)
Sex (male)	80[37.0]	30[41.7]	19[45.2]
TG (mmol/L)	1.96(1.71–2.21)	1.73(1.53–1.88)	1.74(1.57–1.88)
LDL (mmol/L)	3.00(2.56–3.50)	3.29(2.68–3.75)	3.32(2.82–3.75)
HDL (mmol/L)	1.12(0.97–1.25)	1.42(1.36–1.47)	1.43(1.38–1.47)
RBC (10^12/L)	4.54(4.26–4.76)	4.50(4.27–4.79)	4.63(4.39–4.84)
ALP(U/T)	80.27(77.92–82.82)	80.39(77.86–83.03)	81.89(79.27–84.19)
Hemoglobin	135.37(132.60-137.60)	133.86(131.82-135.72)	133.93(132.84-137.46)
Albumin	45.37(42.60–47.60)	46.15(43.21–48.31)	46.27(43.89–48.35)
Creatinine	80.73(75.19–83.22)	82.31(76.43–86.62)	82.54(77.77–86.70)

Data are expressed as medians (quartile ranges) or numbers (percentages)

SONFH = Steroid-induced osteonecrosis of the femoral head; TG = Triglyceride; LDL = Low-density lipoprotein; HDL = High-density lipoprotein; RBC = Red blood count; ALP = Alkaline phosphatase

Imaging histology techniques provide an intuitive and comprehensive understanding of the structural and metabolic status of bone tissue in patients with SONFH. Moreover, the application of transcriptome analysis allows us to more comprehensively parse the biological information related to SONFH at the molecular level. This study aimed to explore the connection between SONFH and lipid metabolism, high-density lipoprotein (HDL), and triacylglycerol (TG) levels by integrating the results of imaging histology and transcriptome analysis. The integration of this biological information is expected to reveal the underlying mechanisms of SONFH development and provide new targets and strategies for prevention and treatment. The in-depth transcriptomic analysis of the molecular mechanisms associated with lipid metabolism in steroid-induced osteonecrosis of femoral head is expected to provide strong support for early diagnosis and individualized treatment of this disease.

## Methods

### Patients

The initial CT data of patients with steroid-induced osteonecrosis of the femoral head necrosis from January 2019 to December 2022 were collected in this retrospective study. A total of 288 ONFH patients from Center 5 were randomly divided into a training group and an internal validation group at a ratio of 3:1 to develop and preliminarily validate the model within the same data source. To further assess the model’s generalizability and robustness, an external validation group of 42 ONFH patients from Center 11 was introduced. The inclusion of this independent dataset aimed to evaluate the model’s performance across different populations, ensuring its reliability for broader clinical application.

**Fig. 1:**
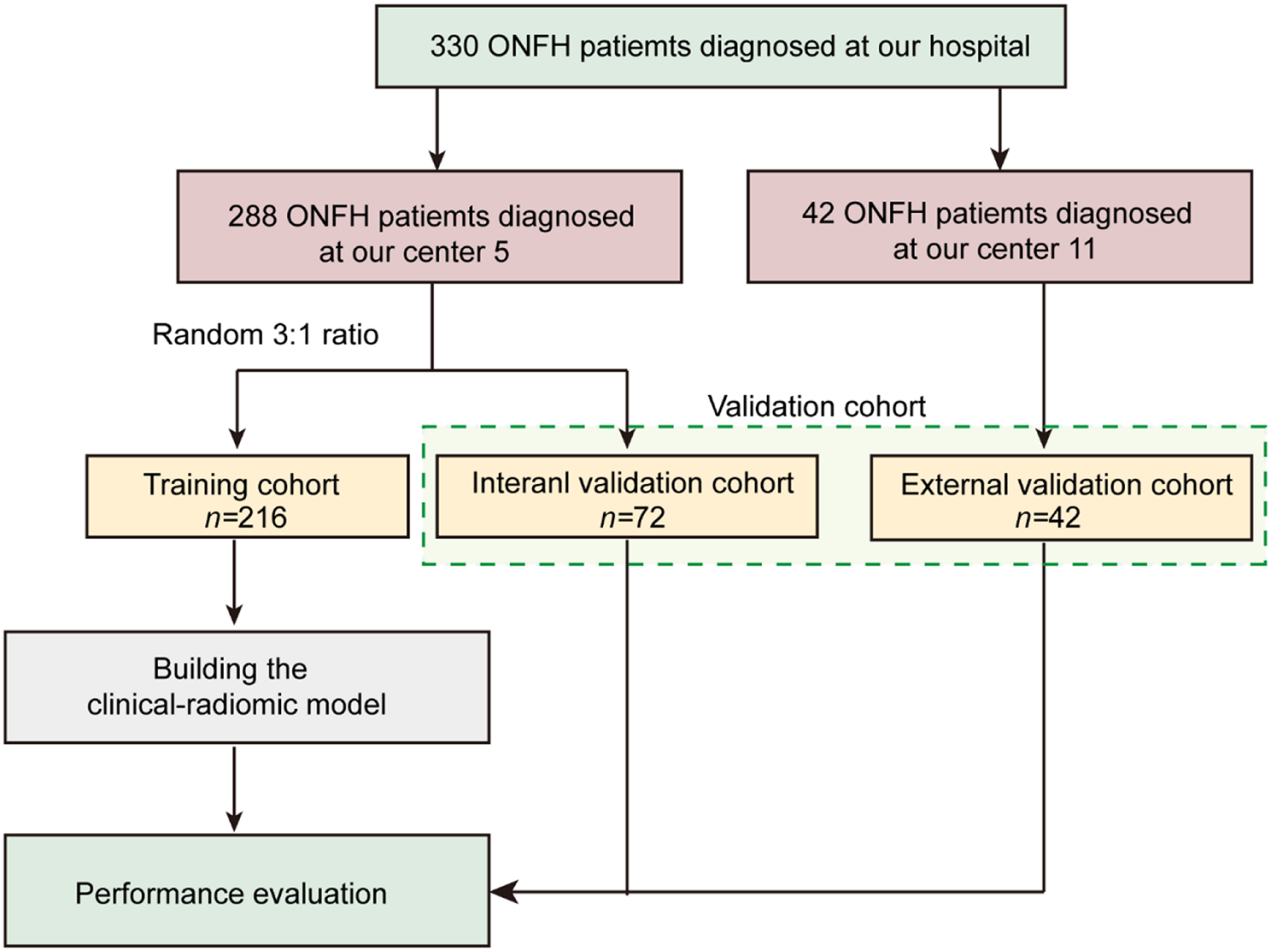
Patient cohort diagram and study methodology. SONFH, Steroid-induced osteonecrosis of the femoral head. The training cohort was used to construct the prediction model, and the validation cohort (the green box) was used to check the accuracy of the model

The inclusion and grouping criteria were as follows: (1) patients who were diagnosed with nontraumatic femoral head necrosis at the initial diagnosis served as the control group and (2) patients with high-risk factors and no clinical symptoms at the time of initial diagnosis (Table 1) but who were diagnosed with nontraumatic femoral head necrosis two years later comprised the test group. Notably, we compared the initial CT data of the two groups. The exclusion criteria were as follows: (1) patients with hip tuberculosis; (2) patients with hip disease due to infection; (3) patients with congenital hip dysplasia; (4) patients with hemophilic arthritis; (5) patients with large osteochondromatosis; and (6) patients aged < 18 years. The flowchart depicting patient inclusion is illustrated in Fig. 1.

### Data collection

In our study, all preoperative CT scans conformed to the guidelines specified in the Chinese clinical recommendations for the diagnosis and management of adult osteonecrosis of the femoral head [15]. Initially, two radiologists systematically gathered images for the primary assessment of femoral head necrosis using the principal imaging traits endorsed by Chen et al. [16] and Cardín-Pereda A. et al. [17].

We subsequently meticulously extracted data from medical records to identify risk factors contributing to femoral head necrosis. The parameters examined included average age, sex distribution, triglyceride (TG) level, low-density lipoprotein (LDL) level, high-density lipoprotein (HDL) level, alkaline phosphatase (ALP) level, red blood cell count, hemoglobin level, albumin level, and creatinine level.

Upon completion, the two coauthors of this investigation independently reviewed all potential patients against the aforementioned criteria. Any disagreements between the two coauthors were harmonized through consensus, with a third coauthor being consulted when necessary.

### Segmenting ROIs and extracting radiomic features

**Fig. 2:**
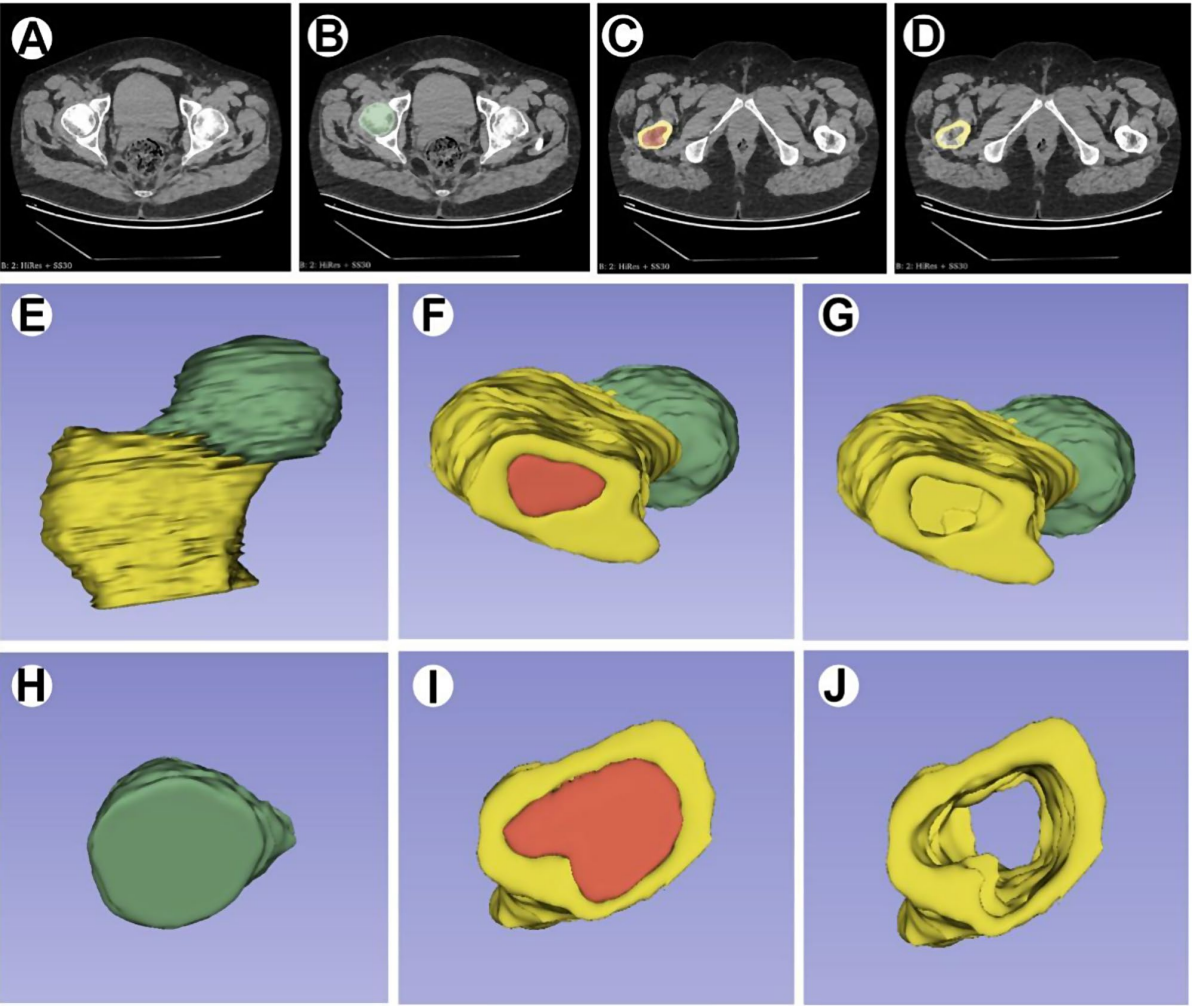
ROI image segmentation analysis. **(A)** Examining the lesion via CT. **(B)** Checking the lesion area on the femoral head (femoral head, green). **(C-D)** Marking the normal area around the femoral head (femur, yellow; bone marrow, red). **(E-G)** Drawing 3D images of the entire diseased femoral head. **(H)** The computer automatically segments these two regions to recognize the features of the diseased femoral head (ROI necrotic femoral head, green). **(I-J)** The computer automatically segments the bone tissue surrounding this femoral head (femur, yellow; bone marrow, red)

Before the segmentation of regions of interest (ROIs) was initiated, a unanimous consensus was achieved among the three collaborating authors regarding the assessment of identical visuals (inclusive of images depicting the target lesion). These regions of interest were subsequently delineated for each specific target lesion by two skilled radiologists, employing open-source software known as 3D Slicer V4.10.2 (accessible at https://www.slicer.org/), which is renowned for its proficient utilization in semiautomatic segmentation procedures. The scope of the designated ROI in this investigation encompassed the entirety of the femoral head section (depicted in Fig. 2). Radiographic attributes were subsequently extracted from every distinct ROI via 3D slicer software, leveraging an enhanced add-on referred to as the “PyRadiomics package” (accessible at https://www.radiomics.io/PyRadiomics.html). This add-on additionally performed the automated extraction of 874 distinct radiographic features from each individual ROI.

### Radiomic signature

In this study, we initially extracted 874 imaging features and identified 268 features that exhibited significant differences between the experimental and control groups. These differences were observed in both the training set and the internal validation set. We employed the widely used least absolute shrinkage and selection operator (LASSO) technique to detect and select the most relevant radiological features. To ensure model robustness and reliability, a 10-fold cross-validation strategy was incorporated into the LASSO algorithm.

Specifically, the LASSO regression process eliminated redundant features that were not relevant to the model’s performance, thereby minimizing any potential adverse effects. In contrast, this method retains imaging features that are significantly associated with steroid-induced osteonecrosis of the femoral head (SONFH) [18]. On the basis of the weight coefficients derived from the LASSO model, corresponding imaging histology labels were constructed. Radiomic scores (Rad-scores) were subsequently calculated for all affected hip cases. These imaging features were selected for their notable differences between the experimental and control groups, and their relevance was further optimized and confirmed through the LASSO algorithm.

### Development of the clinical-radiomic nomogram

The laboratory parameters were arranged according to established standard ranges. A univariate comparison was subsequently conducted on these clinical factors. Significant imaging features were subsequently identified and amalgamated within a multivariate logit model. Employing a column‒line diagram, we visually represented the noteworthy elements earmarked for the multivariate analysis.

### Differential gene screening

Data on osteonecrosis of the femoral head induced by hormones (GSE123568) were obtained from the Gene Expression Omnibus (GEO) database (http://www.ncbi.nlm.nih.gov/geo/). To investigate the differential genes associated with steroid-induced osteonecrosis of the femoral head, we employed the microarray data analysis package (limma) in the R language to identify genes meeting the criteria of |log2-fold change (FC)|>0 and a P value < 0.05 as differentially expressed genes (DEGs). Using 100 linear models from the microarray data analysis package (limma) in R, we selected genes meeting the criteria of |log2-fold change (FC)|>0 and a P value < 0.05 as DEGs. The visualization of these genes with differential expression was conducted via the “xiantao” tool (www.xiantao.love).

### Gene ontology (GO) and KEGG pathway enrichment

In this investigation, we utilized the GO (Gene Ontology) repository to classify the roles of genes into three primary groups: biological processes (BP), cellular components (CC), and molecular functions (MF). These three principal classifications were employed. Simultaneously, we employed the KEGG (Kyoto Encyclopedia of Genes and Genomes) database for in-depth annotation of the differentially expressed genes. These assessments were executed in the R 4.2.1 environment via the clusterProfiler toolkit for enrichment analysis and the org.Hs.eg.db toolkit for ID conversion. Additionally, we visually depicted the outcomes of the GO and KEGG enrichment analyses via the “Xiantao” platform (www.xiantao.love).

### Immune penetration

We accessed the GSE123568 dataset on hormonal femoral head osteonecrosis from the GEO database, comprising 10 normal instances and 30 individuals diagnosed with hormonal femoral head osteonecrosis. Employing the R package “reshape2,” we processed and analyzed the data to compute the prevalence of immune cell penetration in both the normal and steroid-induced osteonecrosis of the femoral head cohorts. A chart illustrating the differences in immune cell penetration between the two groups was generated via the R package “ggplot2.” Spearman’s method was used to assess the connection between immune cells, revealing noteworthy distinctions in penetration prevalence between the two groups.

### Statistical analysis

We conducted a statistical analysis via IBM SPSS software (version 25.0) and the R statistical tool (version 3.3.3, available at https://www.r-project.org). To ascertain variances in clinical factors across the two groups, we employed univariate analysis, utilizing either the chi-square test or Fisher’s exact test for categorical variables. To compare radiomic feature values, we adopted one-way ANOVA. By employing the “glmnet” package, we executed LASSO regression model analysis. The generation of ROC curves involved the utilization of the “pROC” package. The nomogram was constructed through the application of the “rms” package. To assess disparities in AUC values among these models, we employed the Delong test. The Decision Curve Analysis(DCA), however, was executed via the “dca.R.” package. Statistical significance was established at a threshold of *P* < 0.05.

## Results

### Clinical characteristics of the patients

A total of 288 ONFH patients from Center 5 were randomly divided into a training set (216 patients) and an internal validation group (72 patients) at a ratio of 3:1. The training group, comprising 216 patients from Center 5, was used for initial model development and training. An internal validation group, also from Center 5, included 72 patients to preliminarily validate the model’s performance within the same data source. Additionally, 42 ONFH patients from Center 11 were selected as an external validation group to assess the model’s generalizability and robustness on data from a different source (Fig. 1). As shown in Table 1, within the training group, the median age was 44 years, while 80 patients were male. Employing the t test and chi-square test, the training and validation cohorts were assessed, revealing marked disparities in TG levels between these two cohorts. A comparative analysis of the training and internal validation cohorts revealed distinctive discrepancies across four clinical attributes: age, sex, ALP, and hemoglobin. Conversely, the HDL and RBC indices differed between the training and external validation cohorts. Notably, Supplementary Table 1 shows that the remaining clinical attributes did not significantly differ between the training and validation cohorts.

### Radiomic analysis

**Fig. 3:**
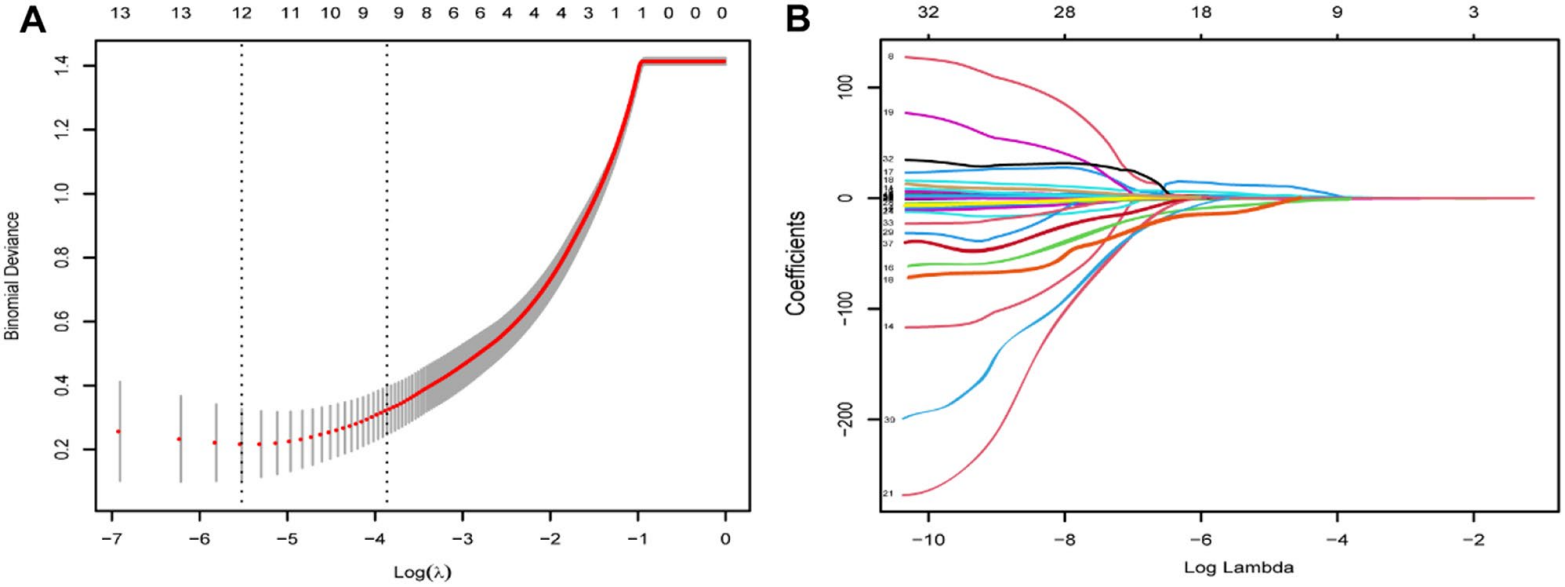
Radiomics signature development. The LASSO algorithm groups radiology characteristics and, based on the minimum criterion of cross-validation, selects the optimal tuning parameters in the lambda model ten times with LASSO. **(A)** Tenfold cross-validation was used in the LASSO model. **(B)** LASSO coefficient profiles of the texture features

By using 3D slicer software, a total of 874 distinct imaging features were extracted from the regions of interest (ROIs) found within 288 CT images. Through a meticulous process of eliminating highly correlated attributes, 268 features were effectively preserved out of the initial pool of 874. The application of the LASSO algorithm subsequently facilitated dimensionality reduction, pinpointing the most essential attributes (as illustrated in Fig. 3A–B). These findings paved the way for the identification of 11 enhanced imaging characteristics distinguishing between SONFH and pre-SONFH patients, as demonstrated in the supplementary data (Supplementary Table 2, Radiology of SONFH patients and pre-SONFH of the femoral head).

### 3. Clinical-Radiomic Nomogram

**Fig. 4:**
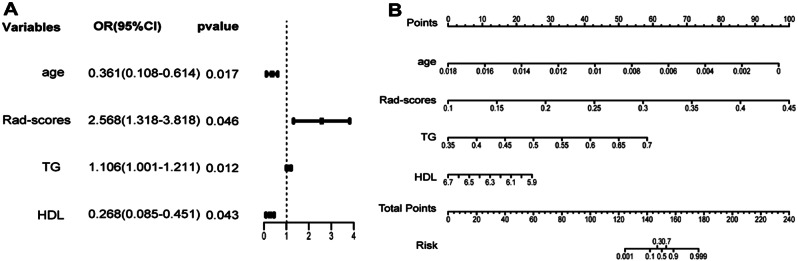
Forest plot depicting significant variables in the model. **(A)** Forest plot depicting significant predictors of objective response via a multivariate regression model. **(B)** A nomogram calibrated according to the coefficient of each factor

Within the training group, several noteworthy clinical variables, namely, age, triglyceride levels (TG) level, and high-density lipoprotein (HDL) level, were examined. Notably, Radiomics scores (Rad-scores) also emerged as statistically significant during univariate analyses, a finding detailed in Supplementary Table 3. By employing regression modeling for multivariate analysis, the combined influence of Rad-scores alongside the aforementioned clinical variables was investigated. This comprehensive approach led to the identification of Rad-scores and the three clinical variables as the optimal predictors, all of which were subsequently incorporated into the final model. The ultimate outcome of this analysis was the creation of clinical radiographic profiles, which are instrumental in predicting the potential susceptibility to femoral head necrosis (as depicted in Fig. 4A–B).

### **4. Predictive nomogram performance**

**Fig. 5:**
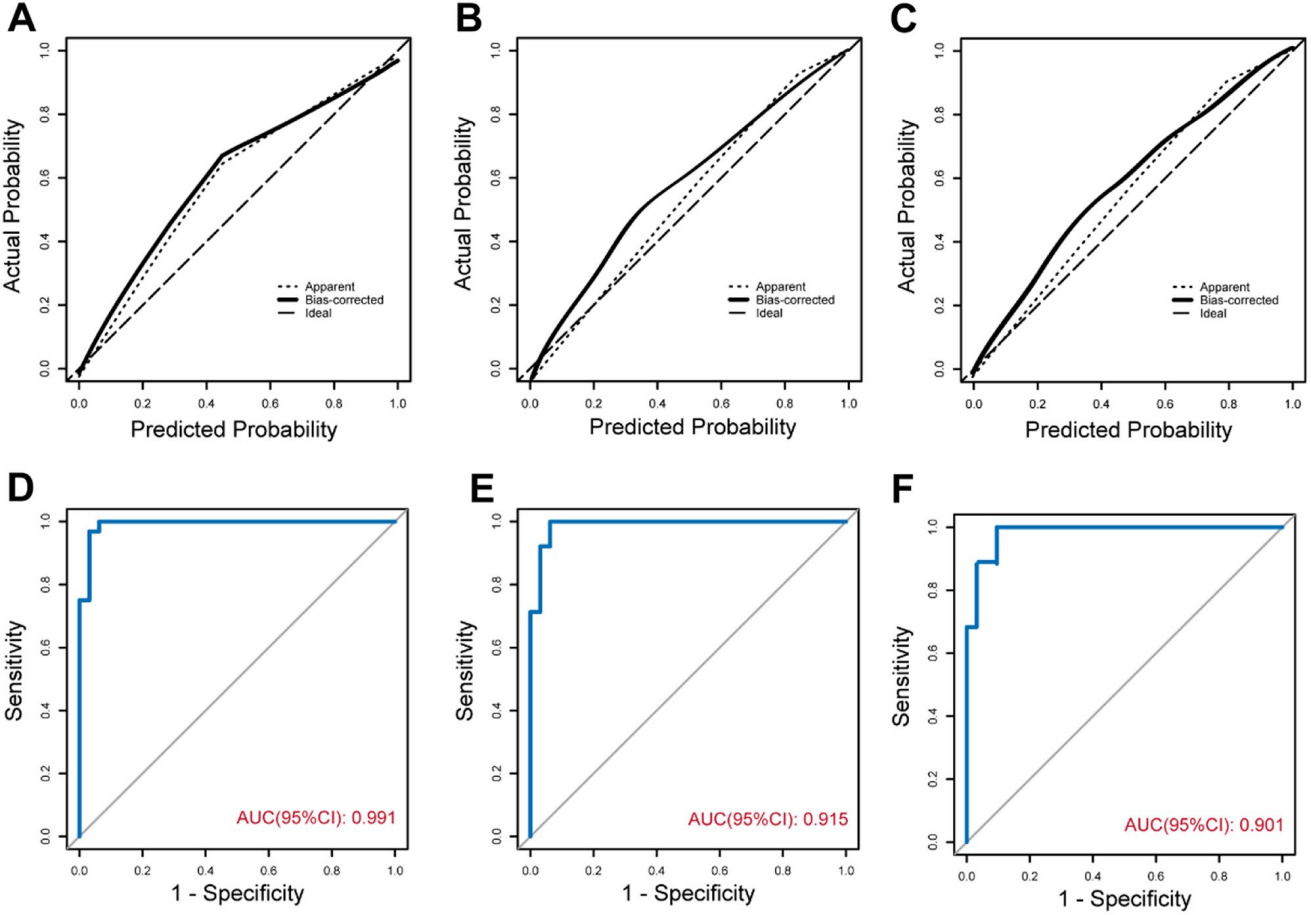
Calibration plot illustrating estimations from the model against the observed probability. Training cohort **(A)**, internal validation cohort **(B)**, and external validation cohort **(C)**. The receiver operating characteristic curves for the training **(D)**, internal validation **(E)**, and external validation **(F)** cohorts. AUC, area under the receiver operating characteristic curve

The clinical radiographic spectrogram efficacy was subsequently assessed via calibration curves and AUC analysis. These curves vividly demonstrate the optimal concordance observed between the prognosticated probabilities derived from the column line diagrams and the factual observations across the three distinct patient cohorts (Fig. 5A–C). The areas under the curve (AUC) pertaining to the training cohort, internal test cohort, and external test cohort were computed as 0.991, 0.915, and 0.901, respectively (depicted in Fig. 5D–F).

### 5. Differential genes and GO and KEGG enrichment pathways

**Fig. 6:**
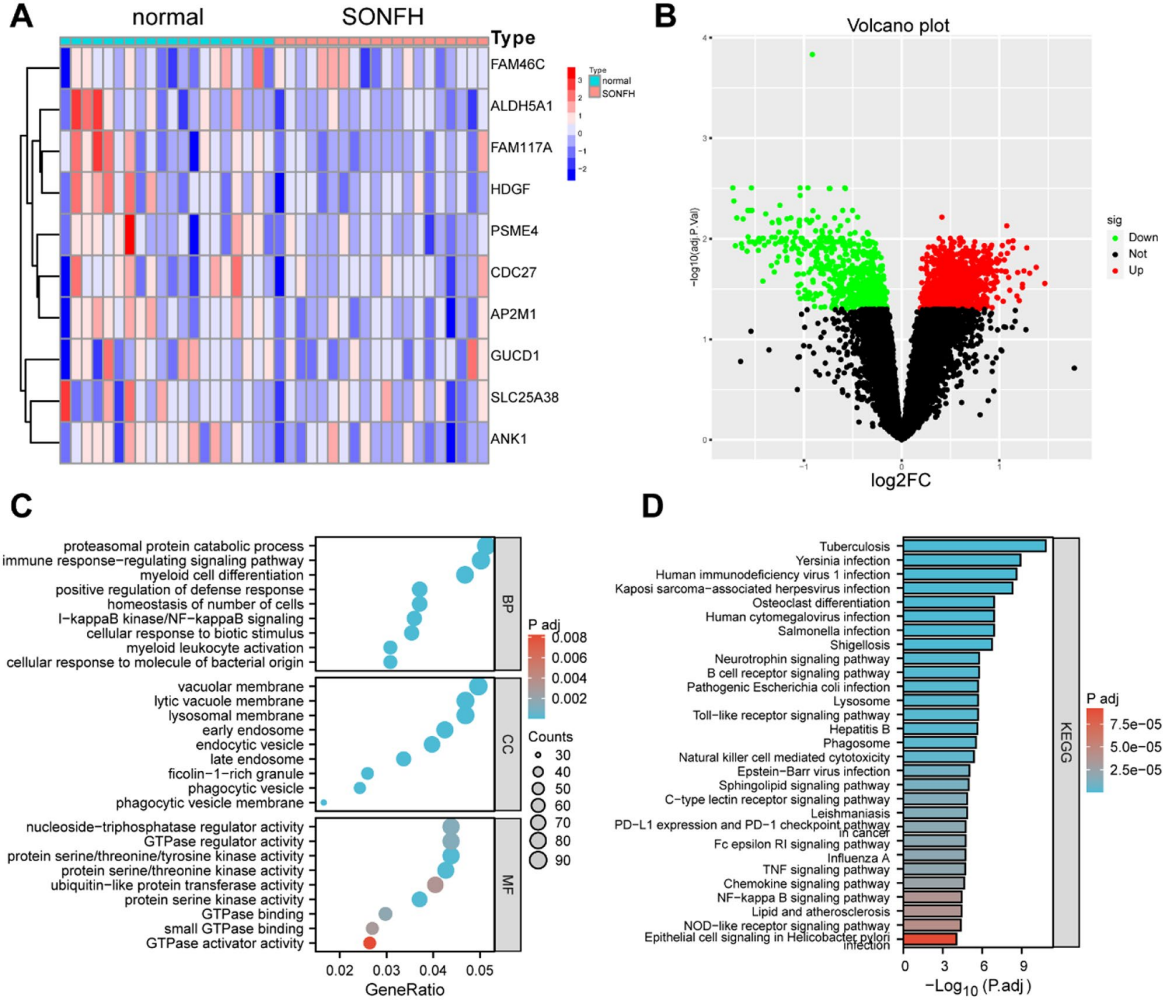
Functional analysis of differentially expressed genes associated with hormone-type femoral head necrosis. **(A)** Difference matrix diagram; **(B)** volcano map; **(C)** GO enrichment analysis; **(D)** KEGG enrichment analysis

**Fig. 7:**
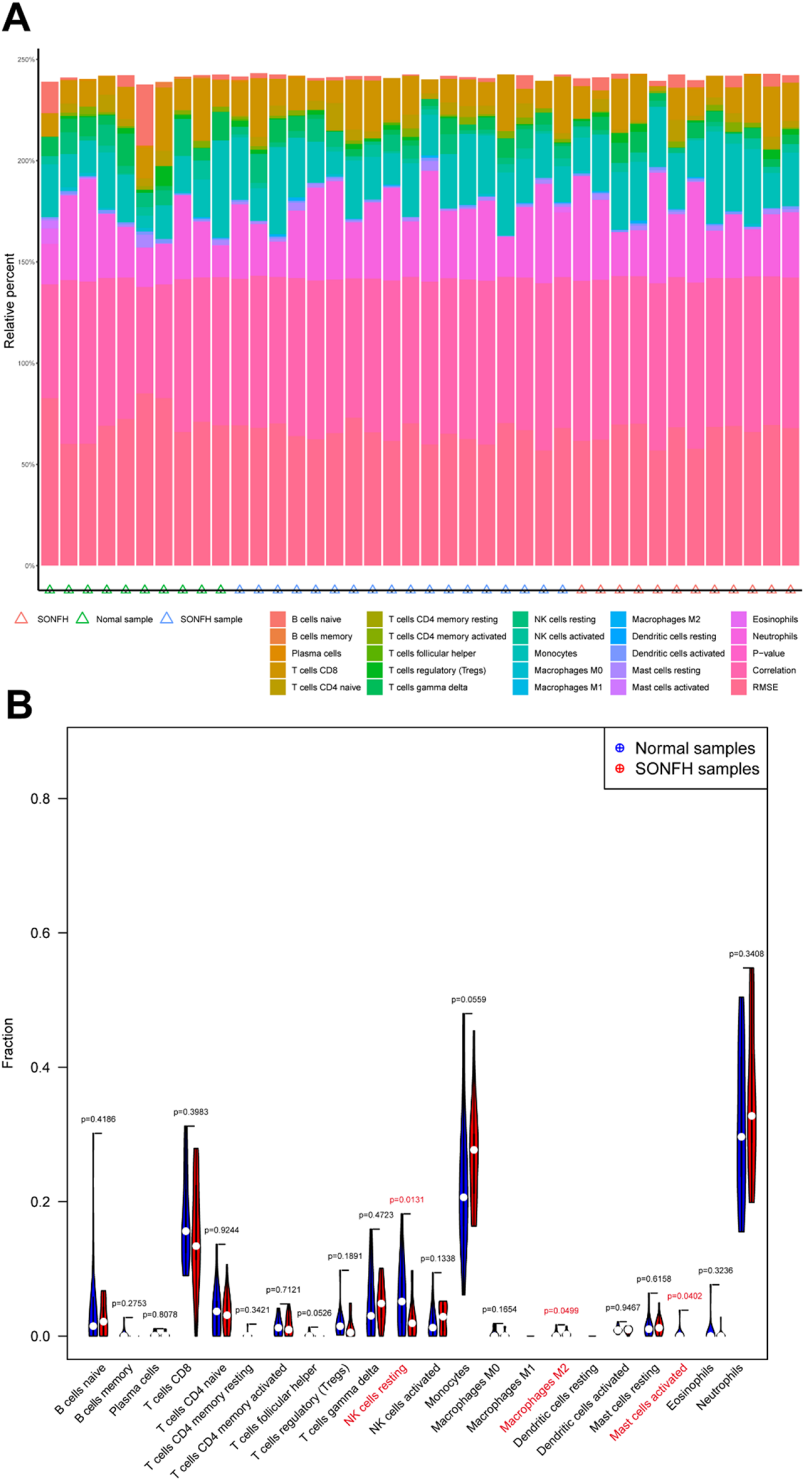
Immunoanalysis of hormone-type femoral head necrosis in the control group. **(A)** Immunoinfiltration map; **(B)** Violin diagram. The red font represents significantly enriched pathways

The top 10 downregulated genes among the differential genes were FAM117A, SLC25A38, HDGF, ALDH5A1, PSME4, ANK1, FAM46C, CDC27, and GUCD1, and the top 10 upregulated genes were SPDYE1, SMPDL3A, ZNF564, KDM5A, SYNGR2, CCNK, HCAR2, CXCR1, and HBP1 (Fig. 6A-B). The major pathways enriched for steroid-induced osteonecrosis of the femoral head-related genes included (Fig. 6C-D) kinase activity, GTPase activity and GTPase binding, phosphatase binding, phosphatase activity and MAP kinase activity, and glycolipid binding, which are primarily involved in the regulation of various functional activities of proteins. These pathways are also indirectly involved in the regulation of lipid metabolism or cellular processes related to lipid metabolism [19]. The sphingolipid signaling pathway and the adipocytokine signaling pathway are involved in various aspects of lipid metabolism and directly participate in the regulation or execution of biological functions associated with cholesterol, HDL and TG [20–23]. Next, we analyzed the relationships between these differential genes and immune cells. Figure 7 A–B shows the relationships between steroid-induced osteonecrosis of the femoral head-related genes and immune cells, which were significantly correlated with resting NK cells, M2 macrophages, and activated mast cells (Fig. 7B). Our findings suggest that these genes may be involved in hormonal femoral progression by regulating multiple immune cells. A review of the literature revealed that M2 macrophages and mast cells were strongly associated with abnormal lipid metabolism [24]. This further supports the accuracy of the clinical variables predicted by our imaging histology; thus, abnormal lipid metabolism can be used as one of the clinical tests for femoral head necrosis.

## Discussion

SONFH is a prevalent and challenging condition within the field of orthopedics and is characterized by its progressive and incapacitating nature. Early and precise diagnosis of SONFH is highly important and is a prerequisite for optimizing joint-preserving measures [25]. Moreover, this diagnostic accuracy also has pivotal implications in terms of conserving medical resources and alleviating the burdens imposed on patients [16, 26]. Importantly, SONFH encompasses an array of pathological transformations stemming from the impediment of the blood supply to the femoral head. This multifaceted etiology underscores the parallelism between SONFH variants induced by diverse factors. The pathological perturbations observed in SONFH patients, stemming from various causal factors, mirror those observed in conventional SONFH patients. The International Society of Bone Circulation (ARCO) and the American Academy of Orthopedic Surgeons (AAOS) jointly delineate SONFH as a condition marked by an interruption or compromise in the femoral head’s vascular supply [27, 28]. This, in turn, precipitates the demise of bone marrow constituents and bone cells, subsequently ushering in structural alterations within the femoral head. This cascade of events culminates, in part, in the collapse of the femoral head and the ensuing impairment of joint functionality. The advent of magnetic resonance imaging (MRI) has facilitated the early detection of SONFH. The availability of MRI has revolutionized the early detection of SONFH, yet the diagnostic landscape remains beset by the challenge of distinguishing SONFH from other hip disorders with similar features. This proclivity toward diagnostic broadening and misattribution primarily stems from a lack of comprehensive familiarity with the unique attributes and diagnostic benchmarks of the SONFH. This finding underscores the pivotal clinical significance of prognosticating femoral head necrosis at an early time point.

In this study, a comprehensive model of SONFH was developed and validated. The model combines three distinct clinical parameters—age, triglyceride levels (TG) level, and high-density lipoprotein (HDL) level—alongside Rad-scores. This composite model was developed to predict the probability of femoral head necrosis. The analysis drew upon retrospective data collected from a cohort of 330 patients across multiple medical centers. Our approach involves the incorporation of markers for SONFH and pre-SONFH. These markers were devised by assimilating diverse radiologic attributes during the course of the study. Their integration played a pivotal role in our SONFH model’s application, enabling more accurate disease likelihood predictions. To initiate the modeling process, we meticulously selected two distinct patient groups. The first group included individuals who were initially diagnosed with necrosis of the femoral head. This group served as the control cohort. In contrast, the experimental group consisted of patients who presented high-risk factors yet remained symptom-free during the initial diagnosis. The subsequent diagnosis of osteonecrosis of the femoral head occurred two years later in this experimental subset. By juxtaposing the initial CT data from both groups, we synthesized a predictive model. Model construction involved scrutinizing the risk factors that strongly correlated with disease onset.

In this study, we employed imaging histology within the realm of the SONFH. Through a sequential process involving sketching, feature extraction, dimensionality reduction, and validation of necrotic regions within CT images, a total of 11 distinct imaging histology attributes associated with pathogenesis were identified. Among these, notable features encompass the following: (1) texture attributes derived from both the grayscale size region matrix and the covariance matrix, which serve as indicators of the grayscale intensity characteristics within necrotic zones and (2) shape characteristics elucidate the three-dimensional dimensions and morphology of the region of interest (ROI), revealing the interplay between the two predominant components shaping the ROI. This set of attributes reveals the intricate correlation connecting the extent, size, and localized signal intensity of the necrotic region to microscopic-level pathological conditions. Previous investigations [29, 30] have highlighted the interrelationship between age and sex and between the selection of anomalous blood markers and susceptibility to SONFH. Additionally, clinical data have underscored the prevalence of SONFH among individuals aged 38–50 years. In our study, univariate Cox regression analysis revealed age, TG, HDL, and Rad-scores as potential prognostic determinants. A multivariate Cox regression analysis further revealed that while age exhibited a moderate correlation with SONFH, TG, HDL, and Rad-scores continued to exhibit a robust correlation, underscoring the intricate connection between lipid metabolism and SONFH. The insights derived from imaging histological attributes also yielded instructive implications for SONFH prediction. Column line graphs revealed discernible patterns, pinpointing advanced age and dyslipidemia as pivotal clinical markers for SONFH diagnosis. These findings support the hypothesis that SONFH disrupts lipid metabolism, increasing vulnerability to this condition. Such disruption may induce anomalies in the femoral artery microvasculature, potentially resulting in the development of lipid emboli within the femoral head. These phenomena collectively perturb blood supply dynamics, ultimately precipitating SONFH. Conversely, an alternate mechanism may involve the proliferation of adipocytes within the bone marrow cavity along with an accumulation of adipose tissue, thereby eliciting heightened pressure within the marrow space. This cascade of events further compromises the blood supply, culminating in the onset of SONFH. In summary, this investigation offers substantial insights into the intricate underpinnings of SONFH through the lens of imaging histology. The cumulative findings not only underscore the role of age, lipid metabolism, and associated clinical markers but also illuminate the multifaceted pathological pathways driving SONFH [31–33]. These findings hold promise for enhancing diagnostic accuracy and shedding light on potential therapeutic interventions, thereby advancing our understanding of this debilitating condition [11].

By utilizing the aforementioned predictors, the generated model exhibits remarkable sensitivity and specificity across both the training and test datasets. Moreover, the training and test sets demonstrated closely aligned AUC values. Quantitative imaging is extensively employed in histological imaging to comprehensively gauge localized necrotic regions at the microscopic scale. These measurements potentially capture intricate cellular-level alterations within necrotic areas, providing intricate insights into their microenvironment. This augmentation complements the perceptual attributes. Conversely, clinical prognosticators evaluate the holistic physical status, lifestyle patterns, and macrolevel biochemical markers of SONFH patients. The model developed within this investigation amalgamates local and comprehensive micro- and macrocharacteristics spanning clinical metrics to imaging attributes. This amalgamation significantly bolsters predictive precision. Additionally, the consolidated model is visually represented through a line graph, facilitating the quantification of predictors. This visualization aids in the prompt detection of SONFH patients in real-world clinical scenarios.

Transcriptome analysis of steroid-induced osteonecrosis of the femoral head differential genes and differential gene enrichment pathway analysis revealed that steroid-induced osteonecrosis of the femoral head, the sphingolipid signaling pathway, the adipocytokine signaling pathway, and other pathways are directly related to lipid metabolism. In addition, these differential genes are significantly correlated with M2 macrophages and mast cell activation, and a review of the literature reveals that these three immune cell statuses affect the level of lipid metabolism [34, 35]. The above results further validate the results of imaging histology, suggesting that there is a strong correlation between the causative factors of steroid-induced osteonecrosis of the femoral head, including lipid metabolism, HDL, and TG, which provides theoretical evidence for the consistency of the results of CT imaging histology.

A potential limitation of this study lies in the exclusive focus on imaging and clinical data without incorporating molecular-level biomarkers, such as genetic or proteomic indicators, which may provide deeper insights into the underlying mechanisms of SONFH. Incorporating these biomarkers in future studies could enhance the model’s precision and reliability. Additionally, this study predominantly utilizes CT imaging for feature extraction, while alternative imaging modalities like MRI, which may reveal different characteristics of early-stage femoral head necrosis, were not included. Expanding the range of imaging techniques may provide a more comprehensive diagnostic and prognostic assessment of SONFH.

However, there are certain constraints within this investigation. First, the study used a retrospective approach, recruiting a limited number of patients at a single center for analysis. Thus, it becomes imperative to validate these findings through subsequent endeavors encompassing multiple centers and a more extensive sample size, lending itself to a prospective design. Second, the absence of an appropriate algorithm for the automated demarcation of necrotic regions in the SONFH led to the adoption of an interactive technique for the manual segmentation of 3D images. This methodology not only demands a substantial allocation of resources but also exhibits a degree of instability. The potential integration of a deep learning network presents an avenue for increasing operational efficiency and mitigating human error. Finally, the scope of this study revolves around the construction of a predictive model for femoral head necrosis, albeit without delving significantly into prognosis-associated questions. The model clearly necessitates supplementation and refinement in subsequent phases, thereby optimizing its utility in facilitating clinical decision-making.

## Conclusions

In summary, the utilization of CT imaging combined with clinical data in crafting a predictive model emerges as a potent, quantitatively visual, and efficacious approach to predicting femoral head necrosis. This approach holds considerable practical importance, offering a novel framework for devising treatment strategies moving forward.

## Electronic supplementary material

Below is the link to the electronic supplementary material.

Supplementary Material 1

## Data Availability

No datasets were generated or analysed during the current study.

## Abbreviations

AUC: Area under the curve
ANOVA: Analysis of variance
DP: Delayed phase
CI: Confidence interval
LASSO: Least absolute shrinkage and selection operator
Nomo score: Nomogram-defined score
Rad score: Radiomics score
OR: Odds ratio
ROI: Region of interest
ROC: Receiver operating characteristic
SONFH: Steroid-induced osteonecrosis of the femoral head
TG: Triglyceride
LDL: Low-density lipoprotein
HDL: High-density lipoprotein
RBC: Red blood count
ALP: Alkaline phosphatase
