# Supplementary material for: Predicting steroid-induced osteonecrosis of the femoral head: role of lipid metabolism biomarkers and radiomics in young and middle-aged adults
Source: J Orthop Surg Res. 2024 Nov 13;19:749. doi: 10.1186/s13018-024-05245-2 (PMC11558989; doi:10.1186/s13018-024-05245-2)
Supplement: Supplementary file 1 — Supplementary Material 1 [file 13018_2024_5245_MOESM1_ESM.docx]

**Supplementary Table 1** Comparison of patient characteristics between the training and validation cohorts. SONFH = Steroid-induced osteonecrosis of the femoral head necrosis; TG = Triglyceride; LDL = Low-density lipoprotein; HDL = High-density lipoprotein; RBC = Red blood count; ALP = Alkaline phosphatase.

|  | Training vs.  Internal Validation (*p* value) | Training vs.  External validation (*p* value) |
| --- | --- | --- |
| Age (year) | 0.005 | 0.175 |
| Sex (male) | ＜0.001 | 0.408 |
| TG (mmol/L) | ＜0.001 | ＜0.001 |
| LDL (mmol/L) | 0.134 | 0.112 |
| HDL (mmol/L) | 0.902 | ＜0.001 |
| RBC (10^12/L) | 0.232 | 0.046 |
| ALP (U/T) | 0.006 | 0.112 |
| Hemoglobin (g/L) | 0.030 | 0.381 |
| Albumin (g/L) | 0.929 | 0.841 |
| Creatinine (µmol/L) | 0.320 | 0.235 |

**Supplementary Table 2** Radiomic feature analysis with LASSO. LASSO = least absolute shrinkage and selection operator; ROIs = regions of interest

| Label name | ROIs | Details of important radiomic features | Coefficient |
| --- | --- | --- | --- |
| Feature 1 | SONFH | Low Gray Level Run Emphasis | 7.7×10^-3^ |
| Feature 2 | SONFH | Dependence Non-Uniformity Normalized | 1.3×10^-3^ |
| Feature 3 | SONFH | Gray Level Variance | 2.9×10^-3^ |
| Feature 4 | SONFH | Cluster Tendency | 5.7×10^-3^ |
| Feature 5 | SONFH | Gray Level Variance | 2.9×10^-3^ |
| Feature 6 | SONFH | Short Run Low Gray Level Emphasis | 1.7×10^-4^ |
| Feature 7 | SONFH | Inverse Variance | 5.0×10^-3^ |
| Feature 8 | SONFH | Gray Level Non-Uniformity Norm | 1.6×10^-3^ |
| Feature 9 | SONFH | Zone Percentage | 4.0×10^-3^ |
| Feature 10 | pre-SONFH | Low Gray Level Zone Emphasis | 2.9×10^-4^ |
| Feature 11 | pre-SONFH | Run Percentage | 3.5×10^-3^ |

**Supplementary Table 3** Univariate and multivariate analyses of radiomic features in the training cohort

| Variables | Univariate analysis | | Multivariate analysis | |
| --- | --- | --- | --- | --- |
|  | *p* value | HR（95% CI） | *p* value | HR（95% CI） |
| Age (≥44,＜44） | ＜0.01 | 0.73(0.68-0.78) | **1** | 0.000012 (0-Inf) |
| Sex (male, female) | 0.51 | 0.87(0.58-1.3) |  |  |
| LDL (≥3.0, ＜3.0) | 0.43 | 0.88 (0.63-1.2) |  |  |
| TG (≥1.96, ＜1.96) | ＜0.05 | 0.47 (0.23-0.94) | **＜0.01** | **7.3 × 10^^19^（1×10^^17^- 5.4×10^^22^）** |
| HDL (≥1.12, ＜1.12) | ＜0.05 | 0.24(0.077-0.78) | **＜0.01** | **0.041 (4×10^-5^-42)** |
| RBC (≥4.54, ＜4.54) | 0.28 | 1.5 (0.74-2.9) |  |  |
| ALP (≥80.27, ＜80.27) | 0.43 | 0.97(0.92-1) |  |  |
| Hemoglobin (≥135.3, ＜135.37) | 0.28 | 1(0.97-1.1) |  |  |
| Albumin (≥45.37, ＜45.37) | 0.28 | 1(0.97-1.1) |  |  |
| Creatinine (≥80.73, ＜80.73) | 0.28 | 1(0.99-1.1) |  |  |
| Rad-scores (Low,High) | 0.047 | 1.4(1-2.1) | **＜0.01** | **0.1 (0.077-0.14)** |
